# Mosquito odour-baited mass trapping reduced malaria transmission intensity: a result from a controlled before-and-after intervention study

**DOI:** 10.1186/s12916-024-03255-9

**Published:** 2024-01-29

**Authors:** Yared Debebe, Habte Tekie, Sisay Dugassa, Richard J. Hopkins, Sharon Rose Hill, Rickard Ignell

**Affiliations:** 1 Department of Zoological Sciences, Addis Ababa University, Addis Ababa, Ethiopia; 2 Public Health Entomology Research Team, Ethiopian Public Health Institute, Addis Ababa, Ethiopia; 3 Aklilu Lemma Institute of Pathobiology, Addis Ababa University, Addis Ababa, Ethiopia; 4 Natural Resources Institute, University of Greenwich, London, UK; 5 Disease Vector Group, Department of Plant Protection Biology, Swedish University of Agricultural Sciences, Alnarp, Sweden

**Keywords:** *Anopheles*, Malaria vectors, Chemical ecology, Malaria prevalence

## Abstract

**Background:**

Conventional vector control strategies have significantly reduced the malaria burden. The sustainability of these methods is currently challenged. Odour-based traps are emerging technologies that can complement the existing tools. Implementation of odour-based traps for mass trapping is limited due to the restricted range of vectors caught with available carbon dioxide-dependent lures, and the lack of comprehensive field studies. The objective of this study was to assess the impact of odour-mediated mass trapping targeting outdoor vectors, using a synthetic cattle urine lure that attracts a wide range of vector species in a variety of physiological states, on malaria prevalence and entomological parameters to determine malaria transmission intensities.

**Methods:**

A controlled before-and-after study was conducted in two rural communities in southern Ethiopia. Baseline monthly entomological and seasonal cross-sectional malaria prevalence surveys were conducted in both communities for a year. Then, mass trapping of mosquitoes was conducted in one of the villages, while the monthly entomological surveillance and seasonal malaria prevalence surveys continued in both villages. Generalised linear mixed models were constructed and tested to determine which factors were significantly affected by the intervention.

**Results:**

Mass trapping contributed to the reduction of the population of the principal malaria vector, *Anopheles arabiensis*, and the associated entomological indicators, the human bite rate (HBR) and the entomological inoculation rate (EIR), in the intervention village compared to the control village. The intervention village had an average HBR by *An. arabiensis* of 3.0 (95% CI 1.4–4.6) during the peak malaria transmission season, compared to 10.5 (95% CI − 0.5–21.5; *P* < 0.0001) in the control village. The intervention village (mean 0.02, 95% CI − 0.05–0.4.8) had a daily EIR eight times lower than the control village (mean 0.17, 95% CI), which likely contributed to the reduced malaria prevalence in the intervention community following its introduction by ca. 60% (95% CI 55–63).

**Conclusions:**

The combined use of odour-based mass trapping and conventional control strategies coincided with a reduction of human-vector contact and malaria prevalence, providing support for odour-baited technologies as a viable option for next-generation vector control tools. Further cluster-randomised control studies are recommended in different eco-epidemiological settings with varying malaria transmission intensities.

**Supplementary Information:**

The online version contains supplementary material available at 10.1186/s12916-024-03255-9.

## Background

The first global malaria response action plan (2008–2015) [1] resulted in a reduction in the malaria burden by ca. 50% [2]. Long-lasting insecticide-treated nets (LLINs) and indoor residual spraying (IRS) have since been the cornerstone of malaria intervention, which rely on the primary vectors displaying intrinsically regulated behaviours of biting and resting indoors [3, 4]. The intense pressure placed on vector populations by the extensive use of LLINs and IRS has, however, led to a change in vector species composition toward those with more opportunistic behaviours [5]. An example of this is the shift in primary vectors in Sub-Saharan Africa from the anthropophilic, indoor feeding and resting *Anopheles gambiae sensu stricto* to the opportunistic, outdoor feeding and resting *Anopheles arabiensis* [6]. Moreover, to fan the flames, a significant proportion of malaria vectors have been reported to bite during the daytime [7]. As a result, time of day and the outdoor environment have become important factors in the increased malaria transmission in Sub-Saharan Africa [8]. Both modelling and direct observational studies have revealed that the addition of targeted outdoor control measures to integrated vector management (IVM) interventions is critical to eradicate malaria [5, 8].

Outdoor feeding and resting mosquitoes are not targeted by LLINs and IRS. While the use of odour-based technologies has been explored as a viable option to target outdoor populations of malaria vectors [9], available synthetic odour blends are, however, generally restricted in their current use for IVM. These blends often target host-seeking females of a limited number of species and require carbon dioxide, which is difficult to procure in affected regions [10]. Moreover, the selection of a trapping system and the proper positioning of the traps within the landscape is required to achieve a high efficacy with the odour-baited traps [11]. Thus, prior to the deployment of odour-baited traps, an in-depth understanding of the spatial ecology of the vector species is needed to provide information on the environmental factors dictating the fine-scale aggregation of different species and physiological states of mosquitoes in the outdoor environment [12–14].

Building on an increased understanding of the ecology of the malaria vectors, Dawit et al. [15] recently identified a synthetic odour blend, which efficiently targets outdoor mosquitoes of various physiological states and species, while not requiring carbon dioxide. This lure relies on the drive of female mosquitoes to obtain nitrogenous compounds from cattle urine to enhance survival, flight and reproduction [15]. In this study, we evaluated the impact of the mass trapping of *Anopheles* mosquitoes on the vector population and malaria transmission in a rural village in southern Ethiopia, using traps baited with a slow-release formulation of the synthetic cattle urine odour blend. For this purpose, monthly entomological and repeated cross-sectional malaria prevalence surveys were conducted to generate baseline information in two rural villages for a year. In the following year, mosquito mass trapping was implemented in one of the villages, while the entomological monitoring and malaria prevalence surveys continued in both villages. For this purpose, Suna traps, specifically developed to target malaria vectors [16], were used, and their placement in the landscape was based on Katusi et al. [14]. The impact of the intervention was assessed by calculating relevant entomological and parasitological parameters in the intervention village compared with the control village. The impact of the intervention and the future use of odour-baited technologies in IVM strategies is discussed.

## Methods

### Study households and participants

The study was conducted in two rural villages of southern Ethiopia, Abulo (6° 03′ 48″ N, 37° 35′ 30″ E) and Magge (5° 51′ 49″ N, 37° 29′ 32″ E), in the Arba Minch Zuria district, Gamo Zone (Additional file 1: Fig. S1). The houses in both villages have a mixture of mud-walled traditional grass-thatched roofs and corrugated iron roofing. A detailed explanation of the study sites is described in Debebe et al. [13]. Abulo, with ca. 900 inhabitants, was selected as the intervention village, whereas Magge, with ca. 700 inhabitants, served as the control. Using a controlled before-and-after study design, the intervention impact of mass trapping on malaria transmission in the two villages was assessed. Monthly entomological monitoring and repeated cross-sectional malaria prevalence surveys were conducted in both villages from January 2018 to December 2019, to generate data on the entomological and parasitological profiles of malaria in the villages, both before and during the intervention.

### Sampling and processing of *Anopheles* mosquitoes

Monthly sampling of *Anopheles* mosquitoes in the intervention and control villages was conducted for 2 years from January 2018 to December 2019. Indoor host-seeking anophelines were sampled using CDC light traps hung 1 m above the ground next to a sleeping person protected by insecticide-treated bed nets (BioQuip Products Inc., CA, USA), while the activity of the outdoor *Anopheles* population was also monitored using CDC light traps placed 50 cm above the ground next to the houses [13]. The CDC light traps for both the indoor and outdoor collections were operated from 18h00 to 06h00. In addition, the indoor resting mosquitoes were knocked down using pyrethrum spray (Mobile^®^, Fujian Quanzhou Gaoke Daily Chemical Manufacturing Co., Ltd., China) [13]. Thirty randomly selected houses from each village, ten houses for each sampling method, were used to monitor the monthly activity of the *Anopheles* populations during the pre-intervention and intervention periods. The collected mosquitoes were morphologically identified using standard keys [17]. Female *Anopheles* mosquitoes were further sorted according to their physiological state as unfed, fed, semi-gravid or gravid following the standard protocol [18]. All *An. gambiae sensu lato* were considered as *An. arabiensis*, as no other members of the species complex have been previously recorded in the area [12]. The presence of *Plasmodium falciparum* and *P. vivax* circumsporozoite proteins in all *An. arabiensis*, *An. pharoensis* and *An. ziemanni* from the indoor CDC light trap collections were assessed using enzyme-linked immunosorbent assay (ELISA) [19].

### Malaria prevalence surveys

To determine the impact of the intervention on the prevalence of malaria, seven seasonal cross-sectional surveys were conducted in both the intervention and control villages throughout the study period: four in the pre-intervention period and three during the intervention period. The surveys were conducted during the long-rain, short-rain and dry seasons. The participants were randomly selected from the general population. The sample size (25%) required for the cross-sectional parasitological examination was determined based on a prior study on the prevalence of malaria in the district [20]. The detailed sampling procedure, preparation of blood smears for microscopy, and treatment of positive individuals is outlined in Debebe et al. [13].

### Mass trapping of Anopheles mosquitoes

After the pre-intervention monitoring, mass trapping of malaria mosquitoes was commenced in the intervention village at the end of the dry season in April 2019. In preparation for the intervention, 50 houses from a total of 134 were selected for trap placement in Abulo in January 2019 (Additional file 1: Fig. S2). Thereafter, 20 W solar panels (Zhejiang Perlight Solar Co., Ltd., Zhejiang, China) were installed on the roof of each house, with a controller (Qingdao Skywise Technology Co., Ltd., Qingdao, China) preventing overcharging of the batteries, installed indoors, connecting the solar panel to a 12-V 18 A h 20 h^−1^ battery (Future Green Technology Co., Ltd., Qingdao, China). The communities were allowed to use the installed solar panels for lighting and phone charging purposes in the early evening (19h00–22h00). Before and during this field trial, several houses in both villages had smaller solar panels installed for similar purposes, suggesting that the installed solar panels had no apparent effect on the overall human behaviours in the villages and thus do not represent a confounding factor in this study. Suna traps (Biogents AG, Regensburg, Germany) were installed outdoors, 20 cm above the ground, next to the wall, away from doors and windows and shaded by the roof, in order to increase the chances of capturing the main malaria vector, *An. arabiensis* [14]. The Suna traps were baited with lures made of low-density polyethylene sachets (100 mm × 100 mm × 0.1 mm) containing high-density polyethylene pellets (2.5 g) loaded, under vacuum, with the synthetic cattle urine odour (2.5 g; Biogents AG) [11]. The Suna traps were operated daily from dusk to dawn, and the lures were replaced every 3–4 weeks to ensure the continuous and consistent rate of release of the synthetic odour blend. To assess the efficacy of the mass trapping, and to monitor the monthly population of mosquitoes affected by mass trapping, sampling of mosquitoes from the 50 Suna traps was conducted for 5 consecutive nights every month. In order to determine the effect of the traps on mosquito catches, a control Suna trap without the lure was run in parallel with the odour-baited traps to assess the effect of the lure. In houses used for monitoring of the mosquito population (some of which had smaller solar panels), lighting was not allowed during the surveillance days to minimise sampling bias, which is known to be affected by light.

### Spatial analysis

The spatial analytical tool, Getis-Ord Gi statistics, was used to analyse the local spatial clustering of malaria vectors and *Plasmodium*-infected individuals following the introduction of the odour-baited traps. Hotspots and cold spots for *Anopheles* mosquitoes and *Plasmodium* parasites were generated in Arc GIS (v. 10.3, ESRI, USA). Then, the impact of the intervention on the spatial clustering was assessed by comparing the results from the hotspot analysis with previous analyses obtained from the pre-intervention study [13].

### Outcome variables

The primary outcomes in measuring the intervention impacts were malaria prevalence, the human-biting rate (HBR) and the entomological inoculation rate (EIR), whereas the density of host-seeking and resting mosquitoes was the secondary outcome. Malaria prevalence per 1000 people was determined by dividing the number of *Plasmodium*-infected individuals by the total number of people tested multiplied by 1000 [21]. The daily HBR was computed for *An. arabiensis*, *An. pharoensis* and *An. ziemanni* by dividing the total number of mosquitoes caught by the total trap nights from the CDC indoor collections for each month [22]. The average daily indoor resting density (IRD) of *An. arabiensis* from the PSC was determined by dividing the total number of mosquitoes knocked down by the pyrethrum spray by the number of houses and collection days in each month [23]. The sporozoite rate (SR) for the three vector species for monthly collections was determined by dividing the number of sporozoite-positive mosquitoes by the number of mosquitoes tested [24]. The daily EIR was estimated from the CDC indoor captures using the formula: 1.605 × (number of circumsporozoite-positive ELISA results from CDC light trap/no. mosquitoes tested) × (number of mosquitoes collected from CDC light trap/number of catches) [25].

### Data analysis

Binary logistic regression was used to predict the probability of a person being infected with malaria in the intervention and control villages during the pre-intervention and intervention periods. The *P*-values were adjusted using the false discovery rate to account for the potential overestimation of the probabilities, which may arise from occasional repeated measures of the same individual. Following the regression analysis, post hoc tests were conducted to test for variation in pair-wise seasonal malaria prevalence in the control and intervention villages pre-and post-intervention. A generalised linear mixed model was used to determine the variation in the mean number of *An. arabiensis*, *An. pharoensis* and *An. ziemanni* collected from all sampling methods by considering seasons and villages as fixed factors and the individual sampling houses as a random factor. Negative binomial regression was used to assess the incidence rate ratios (IRR) of the HBR, SR and EIR in the intervention village, using the control village as a reference category (JMP Pro version 13 SAS Institute Inc., Cary, NC, USA). The impact of the intervention was assessed by calculating the relative percentage of reduction for the primary and secondary outcomes [26] in the intervention village, before and after intervention initiation. The intervention impact was assessed for the two major malaria transmission seasons (the long and short rains). The relative per cent reduction of a given parameter was computed [27] as per cent reduction = 100 (*T*_2_/*T*_1_ × *C*_1_/*C*_2_) − 100, in which *T*_1_ is the parameter in the intervention village during pre-intervention; *T*_2_ is the parameter in the intervention village during intervention; *C*_1_ is the parameter in the control village during pre-intervention, and *C*_2_ is the parameter in the control village during the intervention. Parameters *C*_1_ and *C*_2_ were used as correction factors for each parameter of interest for the intervention village between pre-intervention and intervention.

## Results

### Mass trapping impacts seasonal mosquito density

**Fig. 1:**
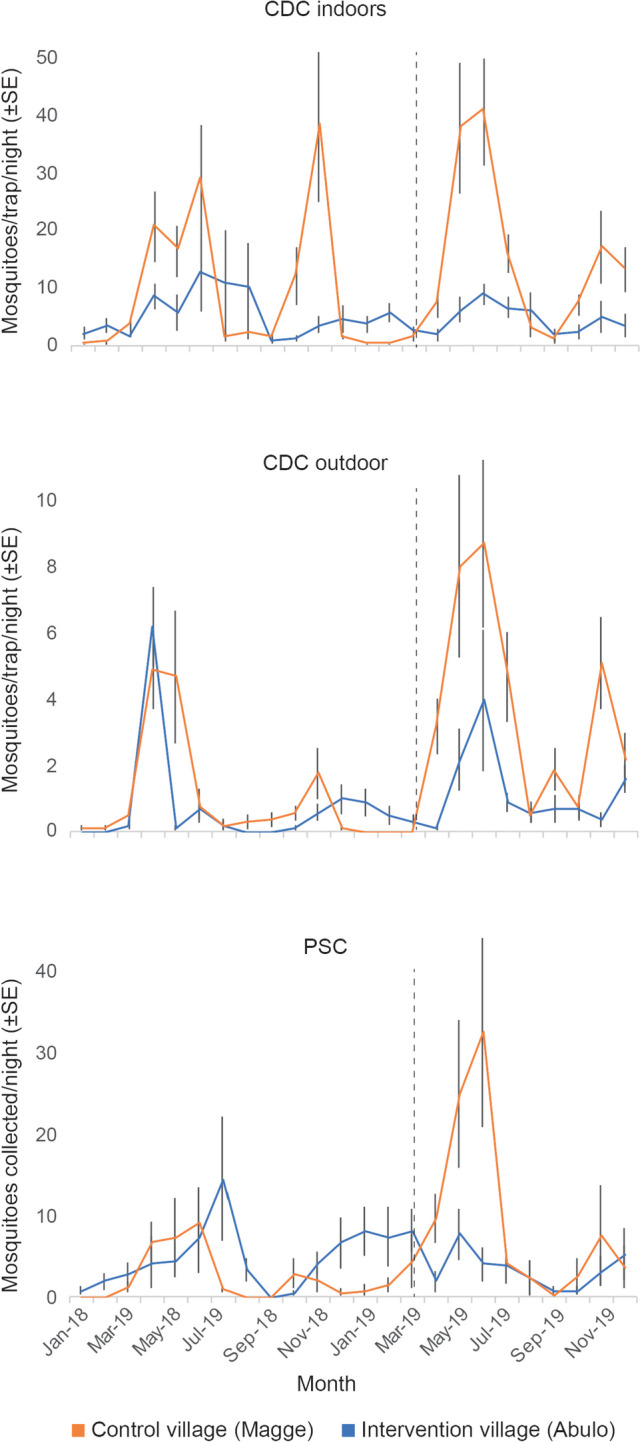
*Anopheles arabiensis* population before and after mass trapping in the intervention and control villages. The activity of mosquitoes indoors and outdoors was determined using CDC light traps, whereas indoor resting mosquitoes were assessed using pyrethroid spray collections (PSC). The dotted line indicates the onset of the intervention

Ten *Anopheles* species (9198 individuals) were captured and/or collected in the study villages during the study. Of these, 97.7% were known malaria vectors, with the primary malaria vector, *An. arabiensis*, as the most abundant species (75.9%), followed by the secondary vectors, *An. pharoensis* (12.7%) and *An. ziemanni* (9.1%) (Additional file 1: Table S1). In general, the activity of indoor and outdoor host-seeking *An. arabiensis*, as well as those resting indoors, during the short and long rainy seasons of the intervention, was significantly higher in the control village compared to the intervention village, whereas the activity in the 2 villages during pre-intervention did not differ significantly (Table 1; Fig. 1). In response to the intervention, with a trap coverage of 37% of households, the surge in the number of active mosquitoes indoors and outdoors was delayed in the intervention village compared to the control village by approximately one month (Fig. 1). Moreover, the relative indoor and outdoor host-seeking activity, as well as the indoor resting behaviour, of *An. arabiensis*, reduced in the intervention village during the long and short rainy seasons, which is in accordance with the hypothesis that the odour-baited traps would reduce the reproductive population of mosquitoes (Table 1; Fig. 1). The numbers of the secondary vectors caught and/or collected during pre-intervention and intervention were low, resulting in increased variance and little to no significant differences between the villages that can be attributed to the intervention (Table 1; Additional file 1: Fig. S3). The majority of mosquitoes caught in the odour-baited traps were host-seeking and blood-fed *An. arabiensis* followed by host-seeking *An. pharoensis* (Additional file 1: Table S2). Monitoring of the control Suna traps resulted in regular catches of zero to few mosquitoes. The IRR of the daily HBR, the sporozoite rate and the seasonal EIR before and after the intervention are presented in Table 2. In addition, data on the efficacy of the mass trapping of malaria vectors is indicated in Additional file 1: Table S3.

**Table 1:** Efficacy of mass trapping of *Anopheles* mosquitoes in indoor and outdoor activities. The relative density of primary and secondary malaria vectors collected/captured per night was reduced in the intervention village following the implementation of odour-baited mass trapping

**Season**	**Collection method**	***Anopheles* spp.**	**Mean pre-intervention density**				**Mean intervention density**				**Relative% red’n (95% CI)**
			**Int. village**	**Con. village**	***F***	***P*-value**	**Int. village**	**Con. village**	***F***	***P*-value**	
Long rains	CDC indoors	*An. arabiensis*	3.2	5.6	1.2	0.30	2	6.9	9.7	0.006	49 (41.3–55.7)
		*An. pharoensis*	1.4	0.5	1.5	0.24	0.4	0.2	2.1	0.16	5.2 (1.4–13.7)
		*An. ziemanni*	0.2	0.3	0.02	0.90	0.3	0.1	6.1	0.02	ND
	CDC outdoors	*An. arabiensis*	1.8	2.7	2.2	0.16	1.6	5	5.6	0.03	54.2 (41.4–59.3)
		*An. pharoensis*	1.6	0.3	0.001	0.97	0.6	0.9	0.2	0.70	88.9 (83.6–94.7)
		*An. ziemanni*	0.7	1.2	0.6	0.44	0.5	0.1	0.03	0.86	ND
	PSC	*An. arabiensis*	7·8	6·2	0·1	0·72	4·1	15	4·4	0.04	78 (74.7–86.4)
Short rains	CDC indoors	*An. arabiensis*	1.4	3.67	1.3	0.27	1.1	3.5	9.4	0.006	16.7 (9.5–21.8)
		*An. pharoensis*	1	1	9.6	0.01	0.4	0.4	1.1	0.32	ND
		*An. ziemanni*	0.5	0.4	1.01	0.34	0.7	0.8	11.7	0.003	43.5 (35.5–47.6)
	CDC outdoors	*An. arabiensis*	0.3	0.6	2.1	0.16	0.9	2.5	5.6	0.03	36 (31.2–43.8)
		*An. pharoensis*	0.9	1.2	0.3	0.62	1.2	1.4	0.1	0.77	ND
		*An. ziemanni*	1	1.2	0.3	0.57	2.9	1.3	4	0.06	ND
	PSC	*An. arabiensis*	3	1.2	2.4	0.14	2.6	3.6	0.1	0.75	71.8

*CDC* Center for Disease Control light trap, *PSC* pyrethroid spray collection, *Int. village* intervention village, *Con. village* control village, *% Red’n* % reduction, *ND* not determined

**Table 2:** Seasonal entomological indices for *Anopheles arabiensis*, pre- and post-onset of intervention. Entomological indices include incidence rate ratios (IRR) of the daily human-biting (HBR) and sporozoite (SR) and the daily entomological inoculation rates (EIR) of the intervention and control villages in each transmission season. The control village was used as the reference category

**Study period**	**Season**	**Entomological indices**	**Village**	**Mean (95%CI)**	**IRR**	***P*-value**
Pre-intervention	Long rains	HBR	Control	8.5 (− 0.7–17)		
			Intervention	4.8 (2.4–7.4)	0.6	0.04
		SR	Control	0.02 (− 0.006–0.04)		
			Intervention	0.02 (− 0.01–0.06)	1.1	0.96
		EIR	Control	0.2 (− 0.1–0.5)		
			Intervention	0.1 (− 0.06–0.3)	0.6	0.76
	Short rains	HBR	Control	5.6 (− 4.34–15.48)		
			Intervention	2.1 (− 0.23–4.41)	0.4	0.07
		SR	Control	0.01 (− 0.014–0.04)		
			Intervention	0.02 (− 0.02–0.07)	1.9	0.94
		EIR	Control	0.07 (− 0.06–0.17)		
			Intervention	0.05 (− 0.1–0.25)	0.7	0.81
Intervention	Long rains	HBR	Control	10.5 (− 0.46–21.48)		
			Intervention	3.03 (1.42–4.63)	0.3	< 0.0001
		SR	Control	0.02 (− 0.002–0.02)		
			Intervention	0.01 (− 0.004–0.02)	0.5	0.93
		EIR	Control	0.2 (− 0.05–0.4)		
			Intervention	0.02 (− 0.01–0.06)	0.1	0.5
	Short rains	HBR	Control	5.2 (− 0.78–11.23)		
			Intervention	1.6 (0.36–0.50)	0.3	0.01
		SR	Control	0.03 (− 0.02–0.07)		
			Intervention	0.01 (− 0.01–0.02)	0.3	0.82
		EIR	Control	0.2 (− 0.1–0.5)		
			Intervention	0.01 (− 0.03–0.1)	0.5	0.5

### Mass trapping of malaria mosquitoes impacts malaria prevalence

**Fig. 2:**
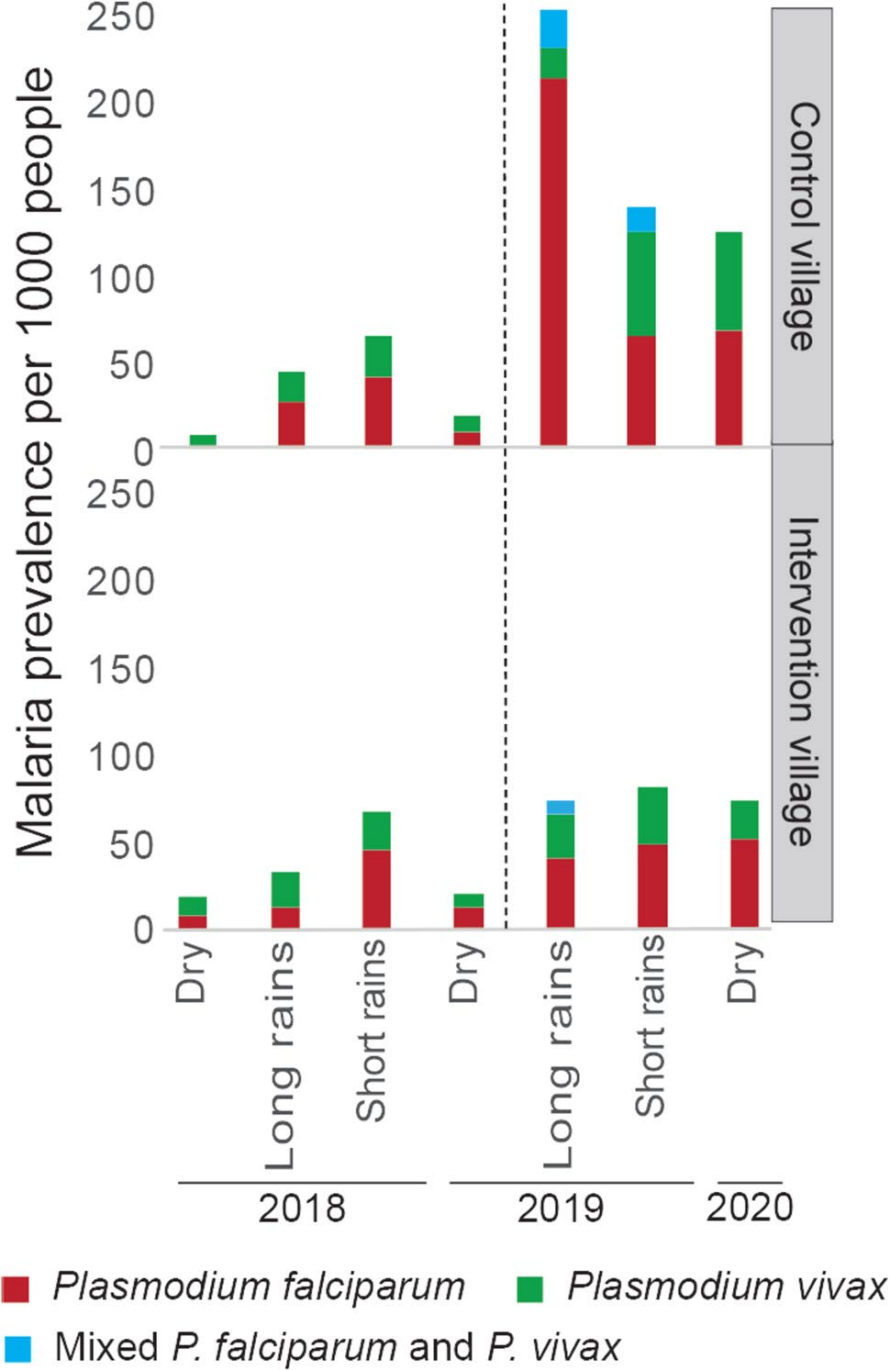
Malaria prevalence in intervention and control villages before and after the implementation of mass trapping. Cross-sectional surveys assessing malaria parasite prevalence were conducted during the long rainy, short rainy and dry seasons. The causative agents for malaria are indicated. The dotted line indicates the onset of the intervention

The probability of a person becoming infected was significantly reduced during the mass trapping in the intervention village (*χ*^2^ = 27.5, OR = 2.5, 95% CI 1.8–3.6; *P* < 0.0001), whereas there was no difference between the two villages during pre-intervention (*χ*^2^ = 0.1, OR = 0.9, 95% CI 0.6–1.5; *P* = 0.71), based on the analysis of the presence of *Plasmodium* parasites in a total of 3229 blood smears. This reduction in malaria prevalence was most evident during the long rainy season when the proportion of people infected with *Plasmodium* parasites in the control village was significantly higher (3.4-fold) than that in the intervention village (*χ*^2^ = 27.1, *P* < 0.0001; Fig. 2). Moreover, the effect of mass trapping was reflected throughout the seasons in the intervention village, with a relative reduction of malaria prevalence in the short and long rains, as well as during the dry season, compared with the control village (Table 3), despite a region-wide increase in malaria prevalence. The incidence of malaria in the district in the 3 years preceding and during the intervention provides the basis for a longer-term comparison (Additional file 1: Fig. S4).

**Table 3:** Efficacy of the mass trapping of *Anopheles* mosquitoes on the seasonal malaria prevalence. Malaria cases per individual tested (prevalence per 1000 people) are reported for the intervention village, in reference to the control village, before and after the intervention

**Season**	**Malaria infection**	**Pre-intervention prevalence**		**Intervention prevalence**		**% reduction (95% CI)**
		**Int. village**	**Con. village**	**Int. village**	**Con. village**	
Long rains	*P. falciparum*	3/240 (12.5)	6/229 (26.2)	10/242 (41.3)	49/232 (211.2)	59% (51.7–64.5)
	*P. vivax*	5/240 (20.8)	4/229 (17.5)	6/242 (24.8)	4/232 (17.2)	− 21%
	Mixed	0	0	2/242 (8.3)	5/232 (21.6)	ND
	Total	8/240 (33.3)	10/229 (43.7)	18/242 (74.4)	58/232 (250)	61% (54.3–66.8)
Short rains	*P. falciparum*	11/237 (46.4)	9/219 (41.1)	11/220 (50)	13/203 (64)	31% (25.7–35.4)
	*P. vivax*	5/237 (21.1)	5/219 (22.8)	7/220 (31.8)	12/203 (59.1)	42% (37–46.8)
	Mixed	0	0	0	3/203 (14.8)	ND
	Total	16/237 (67.5)	14/219 (63.9)	17/220 (81.8)	28/203 (137.9)	44% (40.3–47.6)
Dry season	*P. falciparum*	5/487 (10.3)	2/482 (4.1)	12/228 (52.6)	14/210 (66.7)	68.6% (64.2–72.7)
	*P. vivax*	5/487 (10.3)	4/482 (8.3)	5/228 (21.9)	12/210 (57.1)	69.1% (66.4–73.4)
	Mixed	0	0	0	0	ND
	Total	10/484 (20.5)	6/482 (12.4)	17/228 (74.6)	26/210 (123.8)	63.5% (58.8–67.4)
Overall period	*P. falciparum*	19/964 (19.7)	17/930 (18.3)	33/690 (47.8)	76/645 (117.8)	62.3% (54.3–67.4)
	*P. vivax*	15/964 (15.6)	13/930 (14)	18/690 (26.1)	28/645 (43.4)	46% (42.6–49.5)
	Mixed	0	0	2/690 (2.9)	8/645 (12.4)	ND
	Total	34/964 (35.3)	30/930 (32.3)	53/690 (76.8)	112/645 (173.6)	59.6% (55–63)

*Int. village* intervention village, *Con. village* control village, *ND* not determined

### Impact of mass trapping on the local spatial distribution of malaria vectors and parasites

Local spatial clustering analyses demonstrated a significant reduction in malaria vector densities at the edge and in the centre of the intervention village following the introduction of the odour-baited traps (Additional file 1: Fig. S5). In addition, the formation of a significant cold spot (Gi* *Z* < 1.96, Gi* *P* < 0.05) for vector densities was observed, which was not present before the intervention (Additional file 1: Fig. S5). Cold spots for vector densities in the intervention village overlapped with those for *Plasmodium-*infected people during the intervention period, an effect not indicated pre-intervention (Additional file 1: Fig. S6). In contrast, in the control village, the intensification and expansion of the hotspots (Gi* *Z* > 1.96, Gi* *P* < 0.05) for *Plasmodium-*infected people occurred during the intervention year. Households that were not part of the hotspot pre-intervention were included in the hotspot cluster (Additional file 1: Fig. S6) following the malaria epidemic in the district (Additional file 1: Fig. S4).

## Discussion

The use of outdoor mosquito vector control tools along with established indoor interventions has the capacity to enhance malaria control strategies. In the present study, we demonstrate the potential complementarity of an attractant-driven malaria vector control system with the cornerstone control methods LLINs and IRS in significantly reducing malaria transmission. The reduction of malaria prevalence in this study likely occurred because of a significant suppression of the primary malaria vector, *An. arabiensis*, in the intervention village, despite a region-wide malaria epidemic. These results emphasise that mass trapping, using traps baited with ecologically relevant odour lures that are independent of carbon dioxide, and targeting multiple physiological states of the malaria vectors are a potentially viable option to add to the available tools in the fight against malaria.

Intervention strategies targeting the vector have been the cornerstone of malaria control and are the key focus of global eradication programmes [28]. The intense selection pressures placed on the indoor vector populations by these programmes [29] have led to a shift in focus toward the development of new tools that target vectors in the outdoor environment. Mass trapping of malaria vectors using an attractant-driven control system placed outdoors may significantly reduce vector density (this study, Homan et al. [9]), resulting in a significant reduction in malaria prevalence. The mass trapping system used in this study enabled what appears to be a significant suppression in malaria prevalence in an open rural landscape, representative of common malaria-endemic regions, despite an increase in the overall malaria cases in the region to ca. 35%, with twice the number of malaria cases compared to the three previous years (Additional file 1: Fig. S4). In contrast, the overall annual malaria prevalence in the control village was four times higher during the year of intervention compared to that of the pre-intervention, while the epidemic resulted in a much lower rate of increase (ca. 1.8 times) in the intervention village, likely as a result of the mass trapping.

The implementation of the odour-based mass trapping of malaria vectors in this study coincided with a significant reduction in HBR, SR, and the resulting EIR, and malaria prevalence. Mass trapping has previously been demonstrated to be effective in reducing malaria prevalence, following a significant suppression of host-seeking *An. funestus* [9]. The effects observed in the current study are likely a result of multiple factors linked to the suppression of the local population of the primary vector, *An. arabiensis*. Mass trapping likely resulted in a localised reduction in the density of female *An. arabiensis*, and malaria prevalence within the village landscape, an effect exacerbated at the village edge, a known hotspot for malaria transmission [13]. Moreover, mass trapping coincided with a delayed and attenuated surge in the vector population associated with seasonal change, as seen following IRS treatment [30], compared to the control village and in the intervention village before mass trapping commenced. By co-opting a natural attraction to a supplementary nitrogen source [15], the lure, furthermore, targeted multiple physiological states of the outdoor vector population, thereby enhancing the rate of population reduction in addition to the immediate removal of host-seeking females.

The effectiveness of odour-baited traps depends on other factors besides the ability of the lure to attract the mosquitoes. The positioning of the traps within the landscape highly influences trapping efficacy, as mosquitoes are not evenly distributed even at fine-spatial scales [12, 13]. Fillinger et al. [11] demonstrated that odour-baited traps, used as a component of a push-pull randomised control trial, were ineffective in reducing the human-vector contact outdoors, despite using a lure known to be attractive, implying trap positioning as a main factor for the observed result. In the present study, the placement of the Suna traps used in the mass trapping was based on previous landscape analyses that described the distribution of the malaria vectors in the study villages prior to the implementation of the intervention [12–14], which, in combination with the synthetic cattle urine lure, likely contributed to the observed efficacy of the mass trapping.

Mass trapping using a lure of synthetic cattle urine provides a potentially improved system for vector control and associated malaria reduction, as it does not require carbon dioxide, targets multiple vector species and physiological states as well as being amenable to concurrent use with conventional IVM tools. While assessed in a trap-based system in the current study, the lure may be used to enhance the efficacy of other control techniques, including attract-and-kill or -contaminate and push-pull, thereby potentially removing the need for cost- and labour-intensive trap management [31, 32]. Further testing of the lure in cluster-randomised control trials, either as part of a mass trapping or other initiatives, in other malaria-endemic areas is required to identify the most promising implementation strategy and to assess the social acceptability of the strategy.

## Conclusions

This study demonstrated the potential of the complementarity of outdoor odour-baited traps with indoor vector control methods (LLINs and IRS) in reducing malaria vector density and prevalence. The density of *An. arabiensis* and the associated entomological indices, HBR and EIR, significantly reduced coinciding with a reduction of malaria prevalence in the village where mass trapping was used, along with LLINs and IRS, compared to the control village, where only LLINs and IRS were implemented. Odour-baited traps used in a mass trapping strategy targeting the outdoor mosquito population could be a viable option for vector control, which have the potential to enhance the sustainability of available indoor vector control tools. The use of this odour-based technology, however, requires additional evaluation using more robust randomised control trials in different eco-epidemiological settings, and thus a significant resource commitment.

### Supplementary Information

**Additional file 1:** **Table S1. **Species diversity and abundance of adult female *Anopheles *mosquitoes in the intervention and control villages during the pre-intervention and intervention periods. **Table S2.** Diversity and abundance of mosquito species with different physiological states collected in the odour-baited traps. **Table S3.** Efficacy of the mass trapping of *Anopheles* mosquitoes on the daily human biting (HBR), sporozoite (SR), and seasonal entomological inoculation rates (EIR) of the primary and secondary malaria vectors during the major and minor malaria transmission seasons. **Fig. S1.** Map of the study area. **Fig. S2.** Placement of the Suna traps within the intervention village. The dots represent the geographical locations of each house, while the asterisks represent the locations of the odour-baited traps in the intervention village. **Fig. S3.** Seasonal changes in the *Anopheles pharoensis* and *Anopheles ziemanni* populations before and after mass trapping in the intervention and control villages. The activity of mosquitoes indoors and outdoors was determined using CDC light traps. The dotted line indicates the onset of the intervention. **Fig S4.** Historical data for confirmed malaria cases at the district level in the three years prior to and during the intervention. **Fig S5.** Clustering of malaria vectors generated from hotspot analysis in the control and intervention villages before and after the implementation of mass trapping. **Fig S6.** Clustering of malaria-infected people generated from hotspot analysis in the control and intervention villages before and after the implementation of mass trapping.

## Data Availability

The manuscript and its supplementary files include all data relevant to this study. The raw data is available upon request to the corresponding author.

## Abbreviations

CDC: Centres of disease control
CI: Confidence interval
CPS: Circumsporozoite protein
EIR: Entomological inoculation rate
ELISA: Enzyme-linked immunosorbent assay
HBR: Human biting rate
IRD: Indoor resting density
IRR: Incidence rate ratio
IRS: Indoor residual spraying
IVM: Integrated vector management
LLIN: Long-lasting insecticidal net
OR: Odds ratio
PSC: Pyrethrum spray collection
SR: Sporozoite rate
