# Supplementary material for: Mosquito odour-baited mass trapping reduced malaria transmission intensity: a result from a controlled before-and-after intervention study
Source: BMC Med. 2024 Jan 29;22:41. doi: 10.1186/s12916-024-03255-9 (PMC10823605; doi:10.1186/s12916-024-03255-9)
Supplement: Supplementary file 1 — Additional file 1: Table S1. Species diversity and abundance of adult female Anopheles mosquitoes in the intervention and control villages during the pre-intervention and intervention periods. Table S2. Diversity and abundance of mosquito species with different physiological states collected in the odour-baited traps. Table S3. Efficacy of the mass trapping of Anopheles mosquitoes on the daily human biting (HBR), sporozoite (SR), and seasonal entomological inoculation rates (EIR) of the primary and secondary malaria vectors during the major and minor malaria transmission seasons. Fig. S1. Map of the study area. Fig. S2. Placement of the Suna traps within the intervention village. The dots represent the geographical locations of each house, while the asterisks represent the locations of the odour-baited traps in the intervention village. Fig. S3. Seasonal changes in the Anopheles pharoensis and Anopheles ziemanni populations before and after mass trapping in the intervention and control villages. The activity of mosquitoes indoors and outdoors was determined using CDC light traps. The dotted line indicates the onset of the intervention. Fig S4. Historical data for confirmed malaria cases at the district level in the three years prior to and during the intervention. Fig S5. Clustering of malaria vectors generated from hotspot analysis in the control and intervention villages before and after the implementation of mass trapping. Fig S6. Clustering of malaria-infected people generated from hotspot analysis in the control and intervention villages before and after the implementation of mass trapping. [file 12916_2024_3255_MOESM1_ESM.docx]

**Additional file 1**

Additional file 1.pdf

**Supplementary Tables**

**Table S1.** Species diversity and abundance of adult female *Anopheles* mosquitoes in the intervention and control villages during the pre-intervention and intervention periods.

| ***Anopheles* species** | ***Abulo*** | | | | | | ***Magge*** | | | | | |
| --- | --- | --- | --- | --- | --- | --- | --- | --- | --- | --- | --- | --- |
|  | **CDC light traps** | | | | **PSC** | | **CDC light traps** | | | | **PSC** | |
|  | **Indoors** | | **Outdoors** | |  |  | **Indoors** | | **Outdoors** | |  |  |
|  | **PI** | **INT** | **PI** | **INT** | **PI** | **INT** | **PI** | **INT** | **PI** | **INT** | **PI** | **INT** |
| *An. arabiensis* | 788 | 429 | 108 | 112 | 761 | 307 | 1286 | 1424 | 145 | 351 | 388 | 882 |
| *An. pharoensis* | 381 | 105 | 135 | 77 | 3 | 1 | 225 | 66 | 73 | 102 | 0 | 0 |
| *An. ziemanni* | 120 | 128 | 86 | 143 | 0 | 0 | 94 | 95 | 113 | 56 | 1 | 0 |
| *An. demeilloni* | 19 | 13 | 28 | 4 | 19 | 2 | 21 | 5 | 13 | 1 | 13 | 1 |
| *An. pretoriensis* | 20 | 0 | 39 | 0 | 0 | 0 | 3 | 0 | 0 | 0 | 0 | 1 |
| *An. garnhami* | 1 | 0 | 0 | 0 | 0 | 0 | 0 | 0 | 0 | 0 | 0 | 0 |
| *An. tenebrosus* | 2 | 0 | 3 | 0 | 0 | 0 | 0 | 0 | 0 | 0 | 0 | 0 |
| *An. squamous* | 1 | 0 | 0 | 0 | 0 | 0 | 0 | 0 | 0 | 0 | 0 | 0 |
| *An. cinereus* | 0 | 0 | 1 | 0 | 0 | 0 | 0 | 0 | 0 | 0 | 0 | 0 |
| *An. natalensis* | 0 | 0 | 3 | 0 | 0 | 0 | 0 | 0 | 0 | 0 | 0 | 0 |

PI: pre-intervention period; INT: intervention period; PSC: pyrethrum spray collection

**Table S2.** Diversity and abundance of mosquito species with different physiological states collected in the odour-baited traps.

| **Mosquito spp.** | **Physiological state** | | | | **Total** |
| --- | --- | --- | --- | --- | --- |
|  | Host seeking | Blood fed | Semi-gravid | Gravid |  |
| *An. arabiensis* | 2972 | 314 | 3 | 8 | 3297 |
| *An. pharoensis* | 330 | 7 | 0 | 1 | 338 |
| *An. ziemanni* | 64 | 6 | 0 | 0 | 70 |
| *An. demeilloni* | 1 | 2 | 0 | 0 | 3 |
| *Culex* spp. | 2826 | 221 | 0 | 0 | 3047 |

**Table S3.** Efficacy of the mass trapping of *Anopheles* mosquitoes on the daily human biting (HBR), sporozoite (SR), and seasonal entomological inoculation rates (EIR) of the primary and secondary malaria vectors during the major and minor malaria transmission seasons.

| **Season** | ***Anopheles* spp** | **Entomological indices** | **Pre-intervention** | | **Intervention** | | **% Reduction** |
| --- | --- | --- | --- | --- | --- | --- | --- |
|  |  |  | **Int. village** | **Con. village** | **Int. village** | **Con. village** |  |
| Long rains | *An. arabiensis* | HBR | 4.82 | 8.53 | 3.03 | 10.52 | 49.00 |
|  |  | SR | 0.024 | 0.022 | 0.006 | 0.016 | 59.90 |
|  |  | Seasonal EIR | 14·11 | 22.89 | 3.26 | 26.01 | 79.70 |
|  | *An. pharoensis* | HBR | 2·11 | 0.79 | 0.58 | 0.23 | 5.58 |
|  |  | SR | 0.006 | 0.016 | 0.035 | 0 | -483.33 |
|  |  | Seasonal EIR | 1.586 | 1.586 | 3.213 | 0 | -102.59 |
|  | *An. ziemanni* | HBR | 0.36 | 0·5 | 0.47 | 0.08 | -716.00 |
|  |  | SR | 0.17 | 0.154 | 0.20 | 0.143 | -26.70 |
|  |  | Seasonal EIR | 7.93 | 7·81 | 15.30 | 1.53 | -884.87 |
| Short rains | *An. arabiensis* | HBR | 2.10 | 5.56 | 1.64 | 5.23 | 16.90 |
|  |  | SR | 0.024 | 0.013 | 0.008 | 0.034 | 87.25 |
|  |  | Seasonal EIR | 8.11 | 11.02 | 1.71 | 21.23 | 89.10 |
|  | *An. pharoensis* | HBR | 1.52 | 1.55 | 0.61 | 0·58 | -7.25 |
|  |  | SR | 0.02 | 0 | 0.042 | 0.024 | -110.00 |
|  |  | Seasonal EIR | 4.896 | 0 | 3.294 | 1.586 | 32.72 |
|  | *An. ziemanni* | HBR | 0.80 | 0.56 | 1.03 | 1.27 | 43.20 |
|  |  | SR | 0.095 | 0.304 | 0.16 | 0.075 | -582.67 |
|  |  | Seasonal EIR | 12.393 | 27.693 | 21.35 | 10.86 | -339.30 |

Int. village: intervention village; Con. village: Control village

**Supplementary Figures**

**
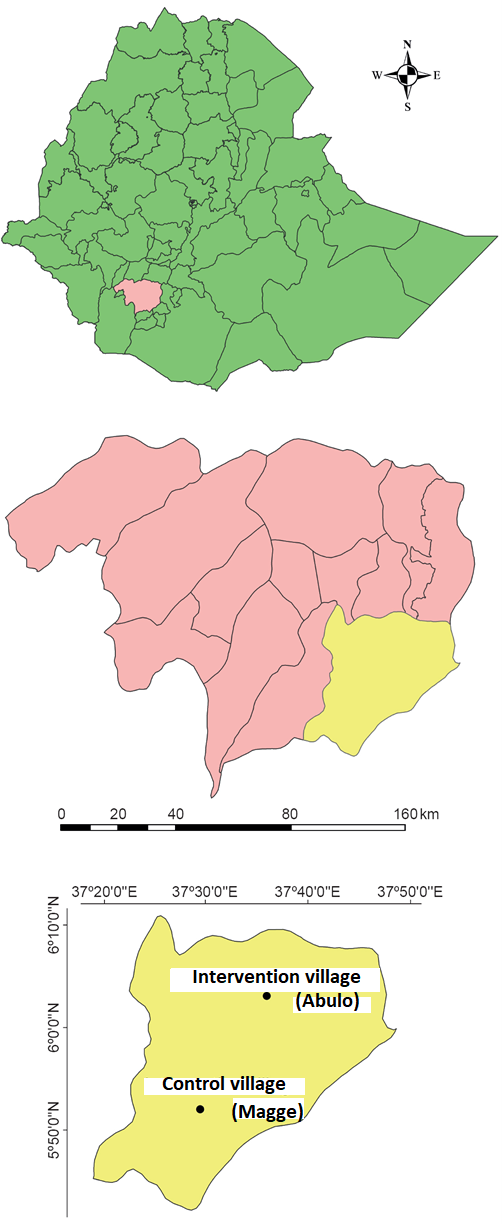
**

**Figure S1. Map of the study area**. The map of Ethiopia (top) with its zonal administration boundaries, indicating the Gamo-Gofa zone (middle), where the study districts are located. The geographical locations of the two study villages, Abulo (the intervention village) and Magge (the control village), are indicated in the bottom map.


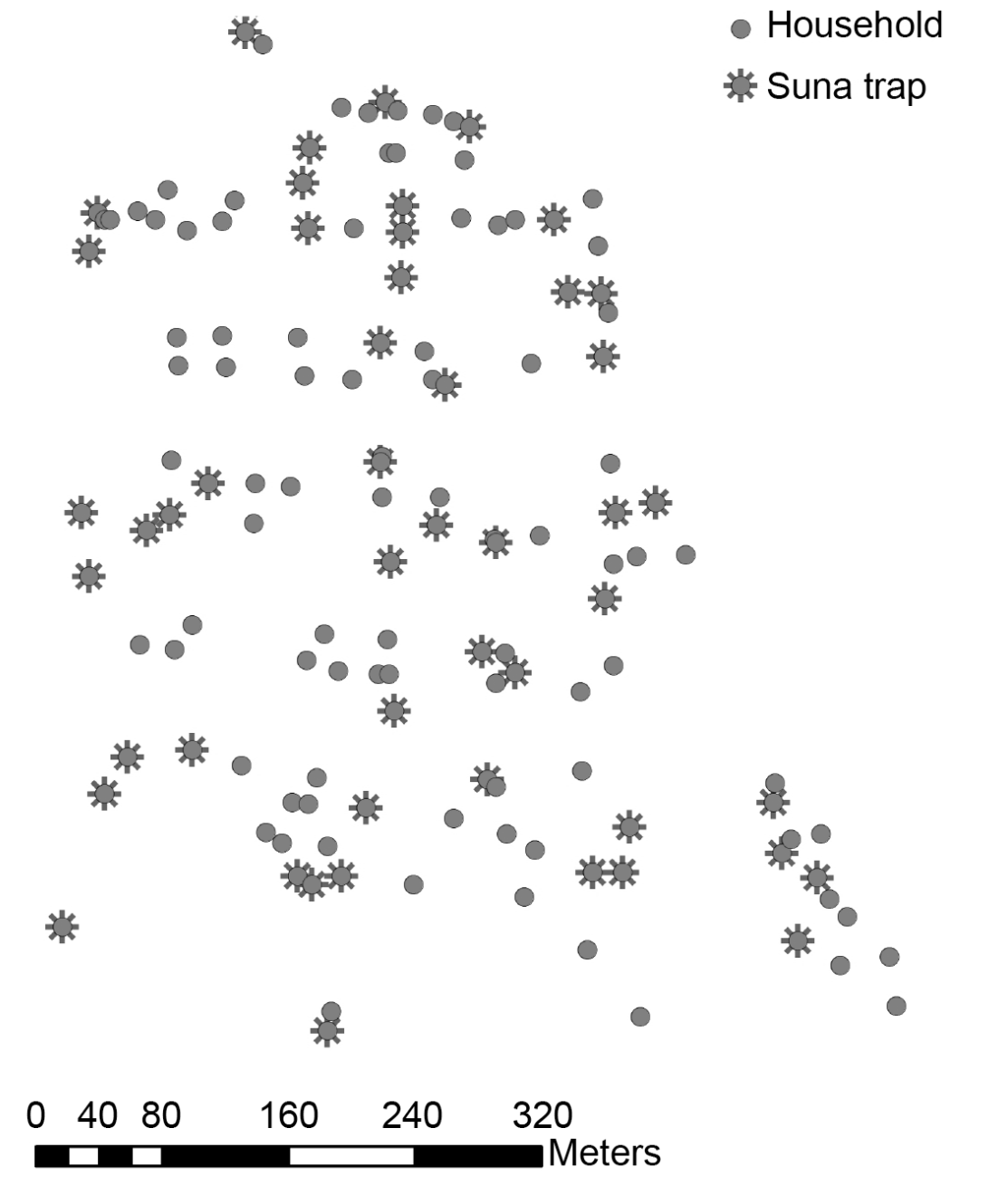


**Figure S2. Placement of the Suna traps within the intervention village**. The dots represent the geographical locations of each house, while the asterisks represent the locations of the houses equipped with odour-baited traps in the intervention village.


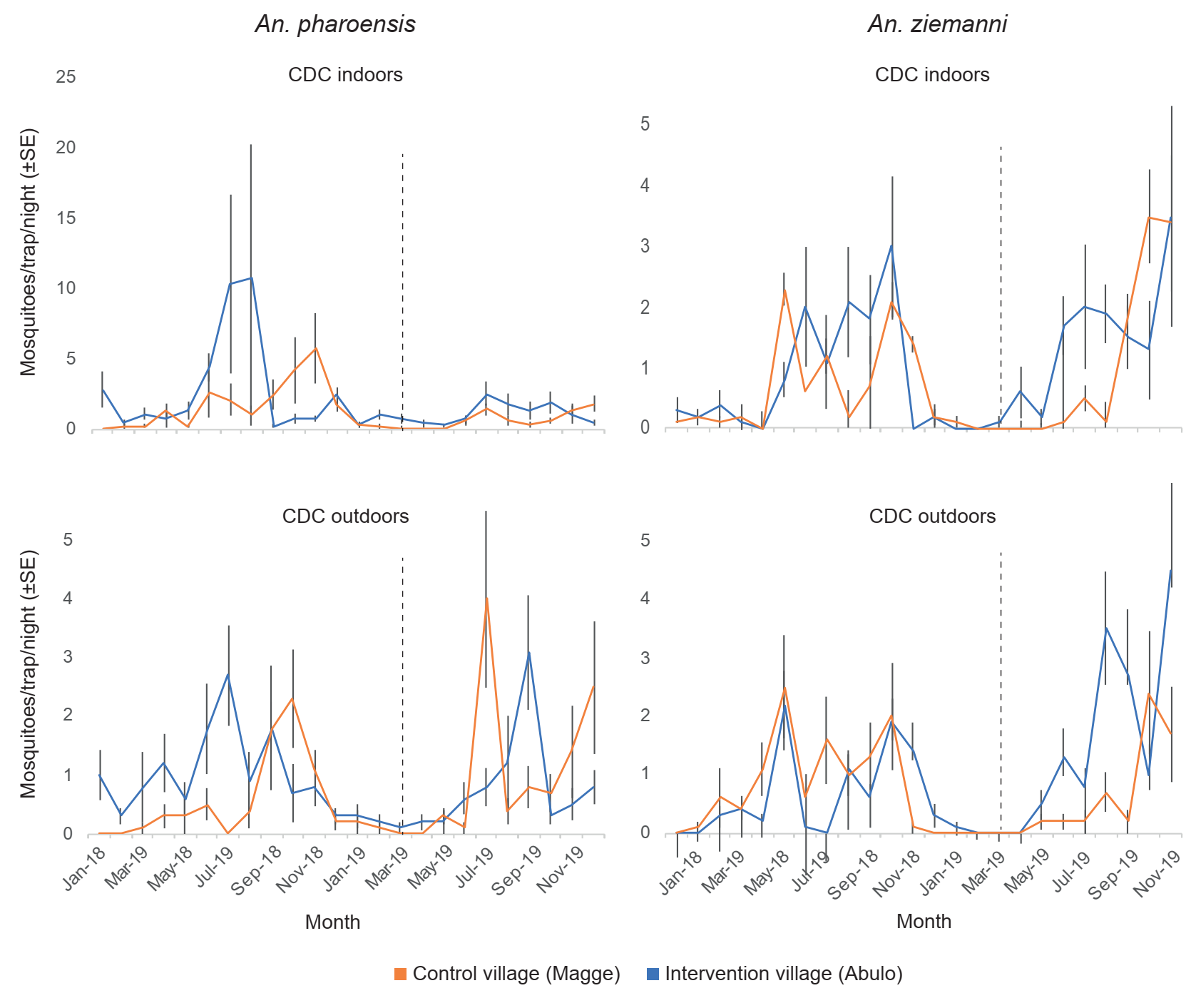


**Figure S3. Seasonal changes in the *Anopheles pharoensis* and *Anopheles ziemanni* populations before and after mass trapping in the intervention and control villages.** The activity of mosquitoes indoors and outdoors was determined using CDC light traps. The dotted line indicates the onset of the intervention.


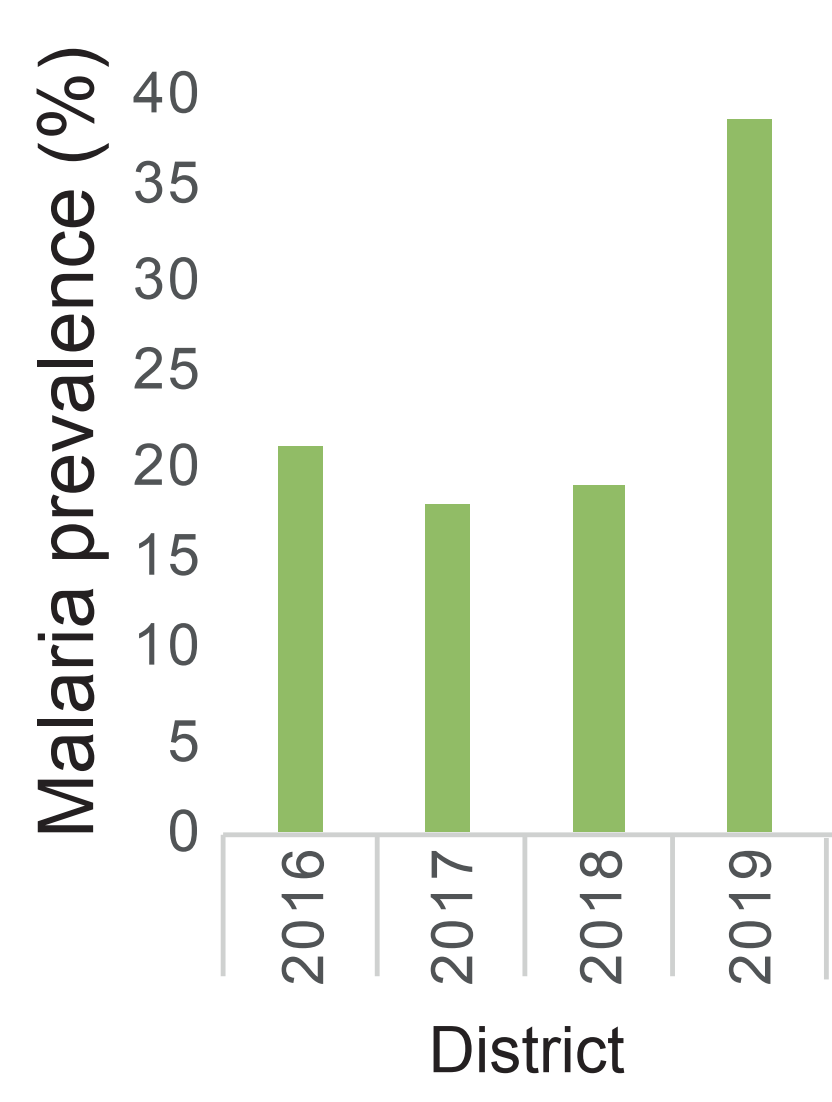


**Figure S4.** Historical data for confirmed malaria cases at the district level in the three years prior to and during the intervention.


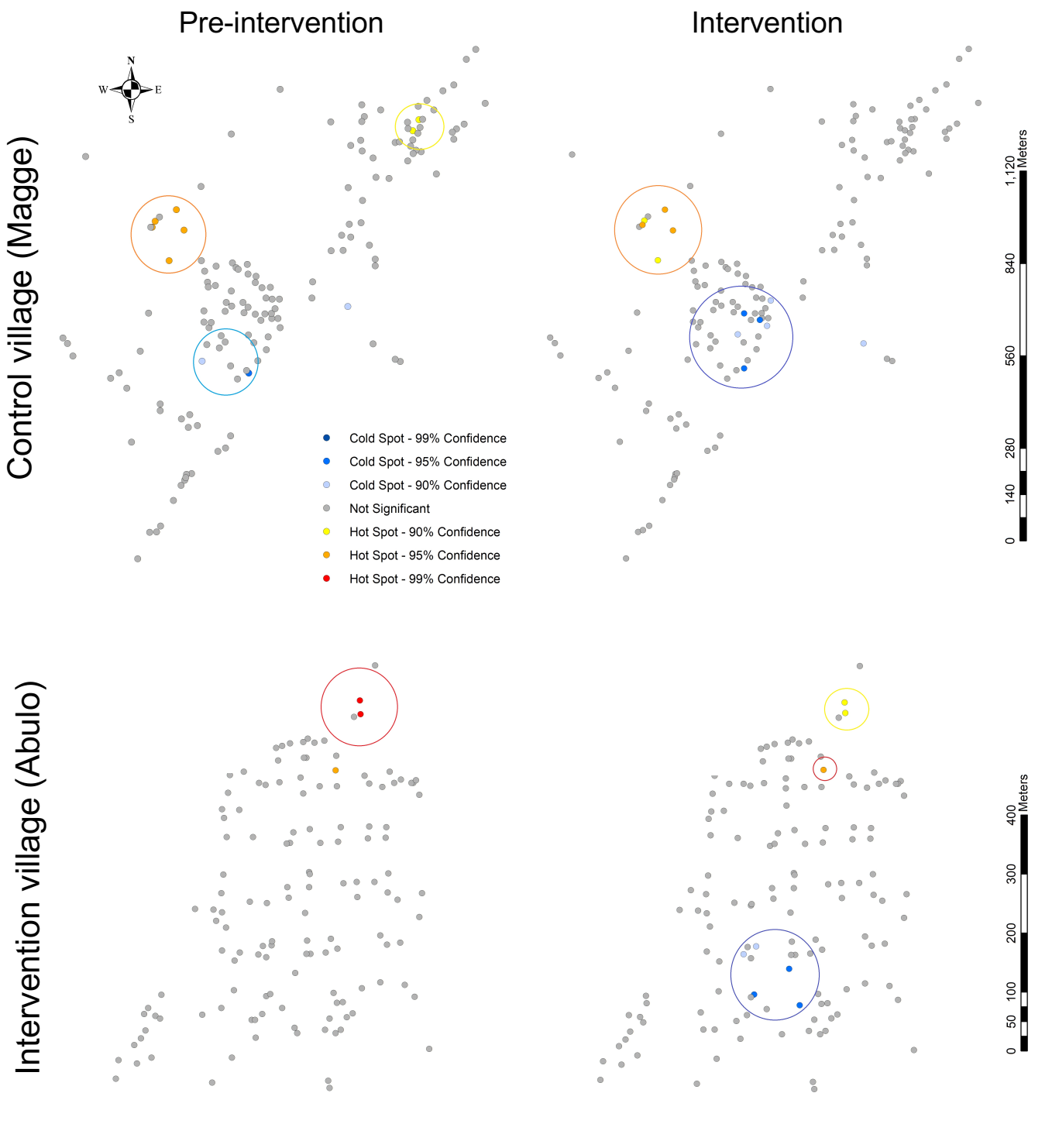


**Figure S5. Clustering of malaria vectors generated from hotspot analysis in the control and intervention villages before and after the implementation of mass trapping.** Hotspots are indicated with 90%, 95% and 99% Confidence intervals (CI). Data from pre-intervention are from Debebe et al. [12].


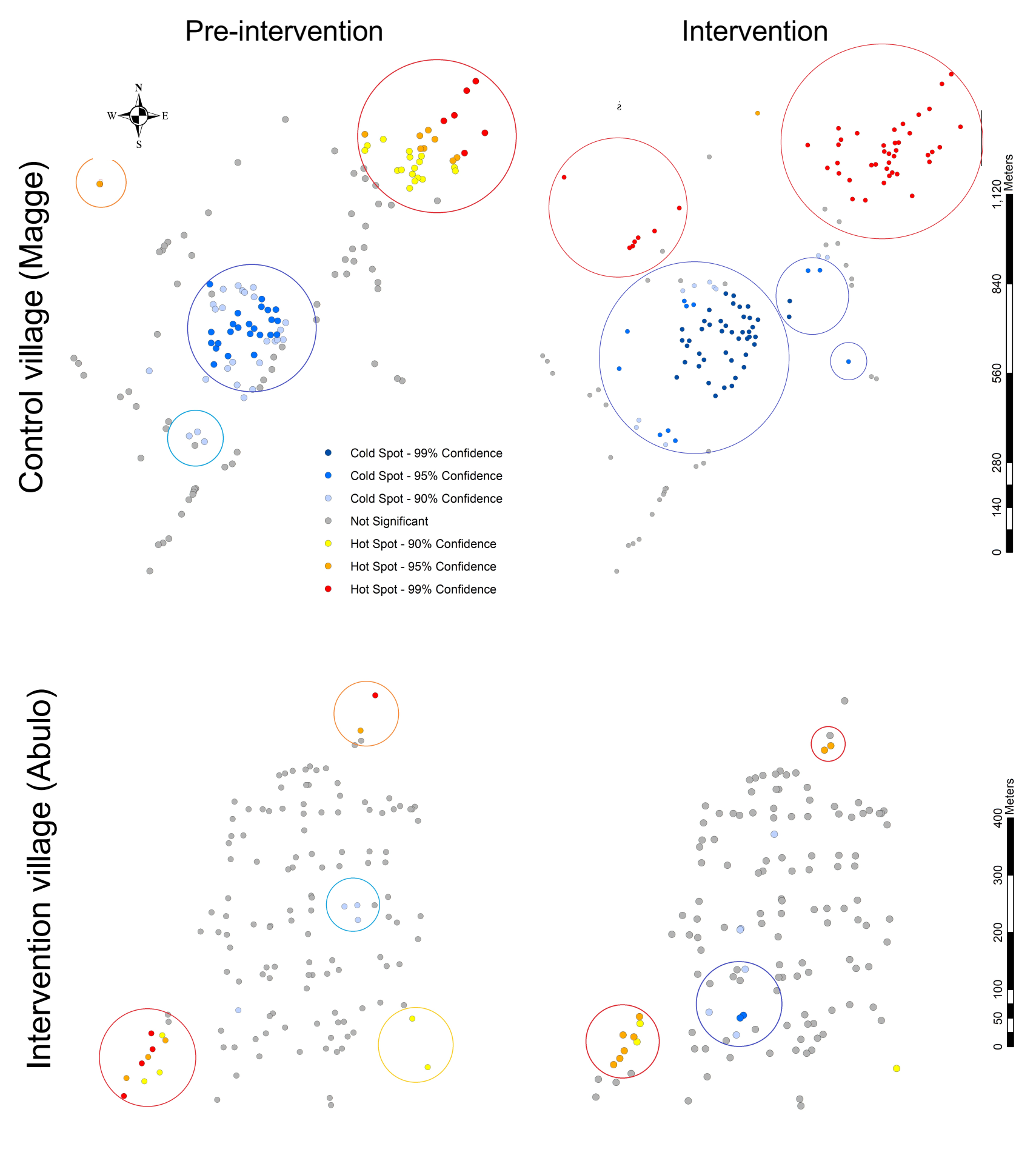


**Figure S6. Clustering of malaria-infected people generated from hotspot analysis in the control and intervention villages before and after the implementation of mass trapping.** Hotspots are indicated with 90% 95% and 99% Confidence intervals (CI). Data from pre-intervention are from Debebe et al. [12].
